# The mechanism of enterogenous toxin methylmalonic acid aggravating calcium-phosphorus metabolic disorder in uremic rats by regulating the Wnt/β-catenin pathway

**DOI:** 10.1186/s10020-025-01067-y

**Published:** 2025-01-22

**Authors:** Xing Fan, Jing Li, Yan Gao, Lin Li, Haisong Zhang, Zhaoyu Bi

**Affiliations:** 1 Department of Nephrology, The Affiliated Hospital of Hebei University, No. 212 Yuhua East Road, Lianchi District, Baoding, 071000 Hebei Province China; 2 Key Laboratory of Bone Metabolism and Physiology in Chronic Kidney Disease of Hebei Province, No. 212 Yuhua East Road, Lianchi District, Baoding, 071000 Hebei Province China

**Keywords:** Enterogenous toxin methylmalonic acid, Uremia, Wnt/β-catenin pathway, Intestinal flora imbalance, Fecal metabolomics, Intestinal permeability

## Abstract

**Background:**

Uremia (UR) is caused by increased UR-related toxins in the bloodstream. We explored the mechanism of enterogenous toxin methylmalonic acid (MMA) in calcium-phosphorus metabolic disorder in UR rats via the Wnt/β-catenin pathway.

**Methods:**

The UR rat model was established by 5/6 nephrectomy. The fecal bacteria of UR rats were transplanted into Sham rats. Sham rats were injected with exogenous MMA or Salinomycin (SAL). Pathological changes in renal/colon tissues were analyzed. MMA concentration, levels of renal function indicators, serum inflammatory factors, Ca^2+^/P^3+^, and parathyroid hormone, intestinal flora structure, fecal metabolic profile, intestinal permeability, and glomerular filtration rate (GFR) were assessed. Additionally, rat glomerular podocytes were cultured, with cell viability and apoptosis measured.

**Results:**

Intestinal flora richness and diversity in UR rats were decreased, along with unbalanced flora structure. Among the screened 133 secondary differential metabolites, the MMA concentration rose, showing the most significant difference. UR rat fecal transplantation caused elevated MMA concentration in the serum and renal tissues of Sham rats. The intestinal flora metabolite MMA or exogenous MMA promoted intestinal barrier impairment, increased intestinal permeability, induced glomerular podocyte loss, and reduced GFR, causing calcium-phosphorus metabolic disorder. The intestinal flora metabolite MMA or exogenous MMA induced inflammatory responses and facilitated glomerular podocyte apoptosis by activating the Wnt/β-catenin pathway, which could be counteracted by repressing the Wnt/β-catenin pathway.

**Conclusions:**

Enterogenous toxin MMA impelled intestinal barrier impairment in UR rats, enhanced intestinal permeability, and activated the Wnt/β-catenin pathway to induce glomerular podocyte loss and reduce GFR, thus aggravating calcium-phosphorus metabolic disorder.

**Supplementary Information:**

The online version contains supplementary material available at 10.1186/s10020-025-01067-y.

## Background

Chronic kidney disease (CKD) is a complicated condition that impacts around 13% of the global population (Evans et al. 2022). Uremia (UR), also namely end-stage renal disease (ESRD), is a serious consequence of CKD characterized by the buildup of substances that are usually eliminated by the kidneys; if left untreated, UR can be fatal (Lim et al. 2021). UR is manifested as the buildup of low molecular weight uremic toxins in the bloodstream, malfunctioning of several organs, and dysregulation of gut microbes; uremic toxins are compounds that cannot be eliminated through urine in ESRD and exert harmful effects (Nigam and Bush 2019). Patients with UR may experience a range of symptoms due to the build-up of metabolic waste, which can affect multiple systems in the body, including the circulatory, digestive, endocrine, and nervous systems. Additionally, patients may exhibit various clinical signs and may also develop complications like calcium-phosphorus metabolic disorder and renal anemia and calcium, seriously impacting the quality of life (Gusev et al. 2021).

Uremic toxins can be classified into two distinct categories including intestinal and endogenous; endogenous metabolites refer to the metabolites that are naturally produced by the human body, like creatinine and uric acid, while enterogenous toxins are primarily categorized based on their origin, either from bacterial metabolism or food consumption (Rysz et al. 2021). Meijers et al. proposed the theory of the “gut–kidney axis” in 2011, suggesting that (Evans et al. 2022) in patients with CKD, the intestinal mucosal barrier is weakened, allowing the absorption of toxic by-products, leading to the development of uremic toxicity and systemic micro-inflammatory response, which consequently speed up the progression of CKD; (Lim et al. 2021) The kidney in patients with CKD is unable to fully metabolize metabolic wastes, such as urea and inosine; the wastes infiltrate the intestinal lumen via the blood vessels in the intestinal wall and change the biochemical conditions of the intestines, which in turn affect the composition and activity of the intestinal microorganisms and trigger the release of harmful substances into the bloodstream, thereby exacerbating renal metabolic burden and CKD progression (Meijers and Evenepoel 2011). In recent years, accumulating studies have verified that the metabolic alterations of gut microbiota in patients with UR may be associated with the increased production of several harmful substances, including phenol, indole, amine, ammonia, and thiol (Rysz et al. 2021; Zhao et al. 2024; Popkov et al. 2022). The enhancement of intestinal barrier permeability in patients with UR facilitates the infiltration of enterogenous toxins into the bloodstream, resulting in the accumulation of toxins in the serum, hence exacerbating kidney disease (Wang et al. 2020).

Methylmalonic acid (MMA) is a dicarboxylic acid predominantly produced as a byproduct of propionate metabolism (Gomes et al. 2020). Also, the alteration of intestinal flora is linked with the change in MA (Tang et al. 2024). MMA is usually associated with gut microbial metabolism. When the composition of intestinal microorganisms alters, normal intestinal metabolites like propionic acid and methylmalonyl-CoA can be converted into MMA under the action of microorganisms (Riphagen et al. 2020). The buildup of MMA in tissues and biological fluids may contribute to the advancement of methylmalonic aciduria and various renal diseases (Galvan et al. 2017). Several factors, such as dietary composition, catabolism, and intestinal microbial production, can impact MMA levels (Riphagen et al. 2020). Reportedly, chronic administration of MMA to rats triggered proteinuria and damage to the renal tubules, accompanied by the enlargement of tubules as well as swelling and disarray of mitochondrial cristae in the epithelium of the tubules (Kashtan et al. 1998). A recent report reveals that the administration of MMA elicited DNA damage in the kidneys of rats (Andrade et al. 2014). Interestingly, serum MMA rises in renal insufficiency (Ganji and Kafai 2018). There is a direct relationship between the level of circulating MMA and All-Cause Mortality in CKD patients, which may predict the adverse prognosis (Wu et al. 2023). However, little is known about the expression of MMA in UR rats and whether it regulates the calcium-phosphorus metabolism disorder in UR rats through the “gut-kidney axis”.

The canonical Wnt/β-catenin pathway is implicated in Renal tubule aging, podocyte injury, and renal fibrosis in diabetic nephropathy (DN), CKD, and acute kidney injury (Schunk et al. 2021; Gong et al. 2021; Chen et al. 2022). Besides, increasing data indicate a close relationship between the Wnt/β-catenin signaling and intestinal flora, the intestinal-brain axis, and the intestinal-liver axis (Spadoni et al. 2016; Ju et al. 2022; Carloni et al. 2021). Moreover, Alisol B 23-acetate modulates the renin-angiotensin system and the “gut-kidney axis”, suppresses the Wnt/β-catenin pathway, and thus mitigates the progression of CKD (Chen et al. 2020). Though the involvement of Wnt/β-catenin in renal injury is well defined, the specific mechanisms that trigger the activation of the Wnt/β-catenin pathway in renal diseases are still not fully understood. In this study, we established a UR rat model and performed 16S rDNA sequencing of the intestinal flora and fecal metabolomics cluster analysis. We further clarified whether the fecal enterotoxin MMA regulated the “gut-kidney axis” through the Wnt/β-catenin pathway in UR rats, which provided a theoretical basis for clarifying the importance of the “gut–kidney axis” in the pathogenesis of UR, and also provided new ideas for the treatment of UR.

## Methods

### Ethics statement

All experimental protocols have been reviewed and approved by the Research and Ethics Committee of The Affiliated Hospital of Hebei University (IACUC-2020XS020), and the approved protocols have been strictly observed. All procedures are in line with internationally recognized animal research guidelines and ethical norms, and all efforts are made to reduce the total number of animals and their suffering.

### Experimental animals

Specific pathogen-free grade Sprague–Dawley rats (male, n = 36, 6 weeks old, weighing 225–250 g) were procured from the Chinese Academy of Sciences (Beijing, China). Rats were housed in an animal room with a temperature of 22–24 °C and a humidity of 40–70% in a 12 h/12 h light/dark cycle and fed a standard laboratory diet (Purina Mills, Brentwood, MO, USA), with free access to water.

### Establishment of UR rat model

The UR rat model was constructed by classical 5/6 nephrectomy according to the method described in the previous literature (Vaziri et al. 2013). Briefly, all rats were fed adaptively for 1 week, and then 18 rats were randomly selected as the Sham group using the random alphabet method, and the remaining 18 received 5/6 nephrectomy, where the upper and lower thirds of the left kidney were resected, and the right kidney was excised one week later. The operation was performed with meticulous attention to hemostasis and adherence to aseptic techniques. The Sham group only accepted bilateral flank incision surgery without nephrectomy. Two weeks post-surgery, serum creatinine (Scr) and blood urea nitrogen (BUN) levels in 18 rats that underwent 5/6 nephrectomy were twice as high as those in the Sham group, indicating the successful establishment of the UR model (Wang et al. 2020). The rat fecal samples from the UR and Sham groups, consisting of 5 rats per group, were separately collected for 16S rDNA sequencing and fecal metabolomics analysis.

### Fecal microbiota transplantation (FMT)

As previously reported, the feces of UR rats were transplanted into Sham rats (Tian et al. 2023). A total of 6 pairs of rats with the same weight were selected, wherein 6 rats were used as fecal donor rats after establishing the UR model, and the other 6 rats after the sham operation were regarded as fecal recipient rats. First, antibiotics were used to clean up the intestinal flora of Sham rats. Broad-spectrum antibiotics containing 1 g/L ampicillin, 1 g/L neomycin, 0.5 g/L vancomycin, and 1 g/L metronidazole were diluted with sterile water. The antibiotics were purchased from MedChemExpress (Shanghai, China). Sham rats were given free access to the antibiotic solution for 14 days, and the antibiotic solution was refreshed every 2 days. The feces of UR rats were gathered, diluted in sterile phosphate-buffered saline (PBS) at 1:5 (W/V), then filtered through three layers of gauze, and centrifuged at 100 *g* for 5 min. Thereafter, the supernatant was centrifuged at 6500 *g* for 5 min. The sediments were retained and resuspended in sterile PBS comprising 20% glycerol, and the concentration was 0.5 g/mL. The fecal supernatant was preserved at − 80 °C for further application. During the 2nd week of surgery, Sham rats were administered broad-spectrum antibiotics for 2 weeks, followed by an enema using 1 mL donor fecal supernatant every 2 days for 2 weeks.

### Animal grouping and treatment

According to the random alphabet method, 36 rats were classified into the following 6 groups (n = 6/group) (Supplementary Fig. 1): (1) the Sham group (sham-operated rats); (2) the UR group (5/6 nephrectomy); (3) the FMT group (Sham-operated rats receiving UR rat FMT); (4) the Sham + MMA group (rats were injected with MMA intraperitoneally (1.67 μmol/g body weight) thrice post operation, with an interval of 11 h, and MMA was ultrasonically dissolved in sterile water (Andrade et al. 2014)); (5) the UR + Vehicle group (post-operative intraperitoneal injection of the same amount of solvent as SAL); (6) the UR + SAL group [post-operative intraperitoneal injection of 8 mg/kg Salinomycin (SAL) (Qu et al. 2015)]. MMA and SAL (the Wnt/β-catenin signaling inhibitor) were purchased from MedChemExpress. After 2 weeks, the body weight of rats was measured, and 24-h urine was gathered using metabolic cages for urinary protein (UPr) quantification. On the next day, 100 μL fluorescein isothiocyanate (FITC)-glucan solution was given to the rats by gavage for 4 h. Then, rats were anesthetized by intraperitoneal injection of 0.3% pentobarbital sodium (50 mg/kg), followed by blood collection (2 mL) from the abdominal aorta, and the serum was isolated for biochemical analysis. Subsequently, rats were euthanized by intraperitoneally injecting with excessive 3% pentobarbital sodium (100 mg/kg). Kidney and colon tissues were immediately collected. Part of the tissues were made into tissue sections for histological staining, and the remaining tissues were made into tissue homogenates for western blot assay.

### Biochemical analysis

The levels of renal function indicators Scr, BUN, and UPr, as well as serum inflammatory factors tumor necrosis factor-α (TNF-α), interleukin-6 (IL-6), and IL-1β were measured by enzyme-linked immunosorbent assay (ELISA). Scr (C011-2-1), BUN (C013-1-1), and UPr (C035-2-1) kits were obtained from Jiancheng Bioengineering Institute (Nanjing, Jiangsu, China); TNF-α (CSB-E11987r), IL-6 (CSB-E04640r), and IL-1β (CSB-E08055r) kits were purchased from Zhongmei Biotechnology (Guangzhou, Guangdong, China). Serum calcium (Ca^2+^) and phosphorus (P^3+^) levels were assayed using a fully automated biochemical analyzer (TBA-120FR, TOSHIBA, Kawasaki, Japan), and serum parathyroid hormone (PTH) level was detected using an electrochemiluminescence immunoassay analyzer (Cobas e601, Roche, Mannheim, Germany). Serum and glomerular tissue MMA (E2091Ge, EIAab^®^, Wuhan, Hubei, China) concentrations were examined by ELISA kits. All operations were executed in complete adherence to the manufacturer’s instructions.

### 16S rDNA sequencing

The microbial diversity of 10 rat fecal samples was examined by 16S rDNA sequencing technology, which was entrusted to Qingke Biotechnology Co., Ltd. (Beijing, China). The microbial genomic DNA was extracted from fecal samples utilizing the QIAAMP Fast DNA Stool Mini Kit (QIAGEN, Hilden, Germany). The 16s v3 + v4_b amplicons were sequenced by Illumina Novaseq PE250. The primers were F: ACTCCTACGGGAGGCAGCA and R: GGACTACHVGGGTWTCTAAT. Referring to the Silva 138 database, the DADA2 method (Callahan et al. 2016) in QIIME2 2020.6 (Bolyen et al. 2019) was used for denoising. Feature information was obtained by splicing the two-terminal sequence and eliminating the chimera sequence. Each amplicon sequence variant (ASV) sequence underwent species annotation to acquire the associated species information and species-based abundance distribution (i.e., ASV_TABLE). The species richness and evenness in the samples and the differences in community structure among distinct groups were analyzed based on ASV_TABLE.

### Fecal metabolomics analysis

Fecal metabolomics analysis was completed by APExBIO Biotechnology Co., Ltd. (Beijing, China). The R XCMS (v3.12.0) software package was applied for peak filtering, detection, and alignment to obtain a quantitative list of metabolites (Navarro-Reig et al. 2015). The public database HMDB (Wishart et al. 2007), massbank (Horai et al. 2010), LipidMaps (Hubbard et al. 2007), mzcloud (Abdelrazig et al. 2020), KEGG (Ogata et al. 1999), and a self-built standard library were employed to identify the metabolites. The qualitative results of the metabolites were obtained. Meanwhile, in the quantitative list, the metabolites detected in the secondary spectrum were matched with the fragment ions of each metabolite in the database, in a bid to attain the secondary identification of metabolites. Principal component analysis (PCA), partial least squares discriminant analysis (PLS-DA), and orthogonal partial least squares discriminant analysis (OPLS-DA) were performed on the sample data using the R software package Ropls (Thevenot et al. 2015). The *p* value was calculated according to the statistical test, the variable projection importance (VIP) was calculated by the OPLS-DA dimension reduction method, and the difference multiple between groups was computed by fold change. Metabolite molecular differences were considered statistically significant when the *p* value was < 0.05 and the VIP value was > 1.

### Histology and immunohistochemistry (IHC)

Kidney tissue and colon tissue were taken following the euthanasia of rats. After conventional fixation, dehydration, and embedding, the tissues were cut into 4 μm thick sections. The double-blind principle was followed for histological examination. The sectioning area and sectioning field were randomly selected, and six fields of view for each rat were randomly selected for subsequent analysis. Hematoxylin and eosin (H&E) staining (Li et al. 2022a) was conducted as per the manufacturer’s manual of the H&E kit (Solarbio, Beijing, China), with an optical microscope (Olympus Corporation, Tokyo, Japan) used to observe and photograph the pathological changes of rat kidney and colon tissues.

Masson staining (Li et al. 2022a) was performed in line with the protocols of the Masson staining kit (AWI0267a, Abiowell, Changsha, Hunan, China). The renal fibrosis alterations were viewed and photographed utilizing an optical microscope. The images were analyzed and fibrosis percentages were calculated using the ImageJ software (version 1.61, NIH Image, Bethesda, MD, USA).

Periodic Acid-Schiff (PAS) staining (Li et al. 2022b) was implemented. Simply put, kidney tissue slices were put in Oxidant at room temperature for 6 min. After being washed, sections were soaked in Schiff’s reagent at room temperature in the absence of light and stained for 15 min. Thereafter, sections were subjected to 1-min staining with Mayer Hematoxylin Solution and 3-s differentiation using Acidic Differentiation Solution, followed by 12-min tap water rinsing to blue. The pictures were processed using ImageJ software to determine the glomerulosclerosis indexes (Wang et al. 2020).

Terminal deoxynocleotidyl Transfer Mediated Nick End Labeling (TUNEL) staining was performed (Chen et al. 2021). The apoptotic rate of renal and colon tissues was assessed via the TUNEL kit (11684795910, Roche, Basel, Switzerland). The nuclei were labeled with 4ʹ,6-diamidino-2-phenylindole (DAPI; Beyotime, Shanghai, China) and observed under a fluorescence microscope (BX53, Olympus). The images were analyzed utilizing ImageJ software.

As for IHC (Li et al. 2022a; Jia et al. 2023), the renal and colonic tissue sections underwent standard procedures of dewaxing, rehydration, antigen retrieval, and inactivation of endogenous enzymes, before incubation overnight with primary antibodies at 4 °C. Sections were rinsed and then cultured in the presence of secondary antibodies for 30 min at room temperature. Sections were subjected to nuclear staining using DAB (Sigma-Aldrich, Saint Louis, MO, USA) and counterstaining with hematoxylin. IHC was conducted utilizing a kit (Zhong Shan Jin Qiao Biotechnology, Beijing, China) in conformity with the provided instructions. The positive site was brownish-yellow, and the analysis of images was implemented by ImageJ software. The percentage of positive cells in the Sham group was normalized. The antibody information is listed in Supplementary Table 1.

### Intestinal permeability detection

As reported previously, intestinal permeability was estimated using fluorescein isothiocyanate-dextran 4 kDa (FITC-dextran) fluorescence labeling method (Zhao et al. 2021). FITC-dextran powder (Sigma-Aldrich) was dissolved in pure water to a concentration of 40 mg/mL. Following fasting for 3 h, rats were given 100 μL FITC-dextran solution by gavage. Afterward, 4 h later, the abdominal aortic blood was gathered and serum was isolated. The FITC-dextran that passed from the lumen into blood was determined on a microplate fluorescence reader (FLX-800; Bio TEK Instruments, Winooski, VT, USA) with 530 nm emission wavelength and 485 nm excitation wavelength using the spectrofluorometry method. FITC-dextran exudation (μg/cm/min) was expressed as intestinal permeability.

### Glomerular filtration rate (GFR)

The assessment of GFR was implemented using FITC-inulin clearance (Kim et al. 2021). GFR was computed utilizing a two-phase exponential decay curve fitted in GraphPad Prism (Qi et al. 2004; Berru et al. 2019), but was not normalized to body weight. Simply put, GFR was determined using the formula GFR = I/(A/α + B/β), wherein I was the number of FITC-inulin administered through the bolus injection, A/B represented the y-intercept values of the two decay rates, and α and β were indicative of the decay constants for the distribution and elimination phases, separately (Qi et al. 2004).

### Cell culture and treatment

Rat glomerular podocytes (u004, Yaji Biotechnology, Shanghai, China) were cultivated in EliteCell primary epithelial cell medium (PriMed-EliteCell-001, ICell Bioscience, Shanghai, China). The podocyte-specific marker Wilms tumor 1 (WT-1) was detected by immunofluorescence. The cell purity was higher than 98%, and the cell activity was good. According to the visual observation and transmission electron microscopy examination, cells did not contain HIV-1, HBV, HCV, mycoplasma, bacteria, yeast, or fungi. Cells were categorized into the following groups: Control group, MMA group (treatment with 0.5, 1.0, 1.5, 2.0, 3.0, and 5.0 mmol/L MMA for 24 h), MMA + SAL group, and MMA + Vehicle group (treatment with half maximal inhibitory concentration (IC50) concentration of MMA and 10 μM SAL or equivalent solvent for 24 h (He et al. 2019)).

### Toxicity test and IC50 calculation

Cells were treated with different doses of MMA (0.5, 1.0, 1.5, 2.0, 3.0, and 5.0 mmol/L), and the toxicity was determined by 3-(4,5-dimethyl-2-thiazolyl)-2,5-diphenyl-2-*H*-tetrazolium bromide (MTT) assay. The optical density (OD) value was ascertained at 450 nm using a microplate reader (Thermo Fisher Scientific, Waltham, MA, USA). Specific operations were conducted precisely under the instructions provided in the kit. The IC50 values of glomerular podocytes in rats treated with MMA were calculated.

### Flow cytometry

Cell apoptosis was evaluated through the Annexin V-FITC apoptosis detection kit (BD Biosciences, Franklin Lakes, NJ, USA) and the fluorescence-activated cell sorting (FACS) Calibur™ flow cytometer (BD Biosciences).

### Western blot

Total protein of tissue homogenate and total podocyte protein were extracted on ice using radioimmunoprecipitation assay lysis buffer (P0013E, Beyotime). Nuclear and cytoplasmic proteins were extracted in line with the instructions of the nuclear and cytoplasmic protein extraction kit (P0028, Beyotime). After transfer and blocking, the proteins were cultivated with primary antibodies overnight at 4 °C. The samples were washed and then incubated with a secondary antibody at room temperature for 2 h. β-actin served as the internal reference for cytoplasmic protein, while Lamin B was regarded as the internal reference for nuclear protein. The chemiluminescence kit (ECL Plus, Life Technology) was utilized for the detection of protein bands, followed by gray analysis using Image J software. The details regarding antibodies are shown in Supplementary Table 1.

### Cell supernatant determination

The cell supernatants of each group were collected. According to the manufacturer’s instructions, The levels of TNF-α (CSB-E11987r), IL-6 (CSB-E04640r), IL-1β (CSB-E08055r), and Collagen IV protein (E-EL-R3009, Elabscience, Wuhan, Hubei, China) in the cell supernatants were detected by the ELISA test kit.

Matrix metalloproteinase-2 (MMP-2) activity in cell supernatant was evaluated using the MMP-2 activity detection kit (GenMed Scientifics, Arlington, MA, USA) (Yao et al. 2015). The OD value was assessed by a microplate reader at an excitation wavelength of 330 nm and an emission wavelength of 400 nm.

### Immunofluorescence

Renal tissue sections or podocytes were routinely fixed, washed, and blocked, before incubation with primary antibodies at 4 °C overnight. After sample rinses, the samples were incubated with appropriate secondary antibodies at room temperature for 2 h. Thereafter, the nuclei were re-stained with DAPI for 5 min, dehydrated with gradient alcohol, and the stained sections were visualized by fluorescence microscopy. Image analysis was performed using ImageJ software. The percentages of positive cells in the Sham or Con groups were normalized. Antibody information is shown in Supplementary Table 1.

### Statistical analysis

All data were statistically analyzed and plotted using GraphPad Prism 9.5.0 (GraphPad Software, San Diego, CA, USA) and R software (Version 3.5.3). The normal distribution was tested by the Shapiro–Wilk test. Measurement data conforming to the normal distribution were expressed as mean ± standard deviation (SD), compared between two groups using the independent sample *t-*test, and compared among groups using one-way analysis of variance (ANOVA), followed by Tukey’s multiple comparison test. Measurement data of non-normal distribution were expressed as median (minimum, maximum) values and analyzed using the Mann–Whitney U test between two groups. The ACE index reflected microbial flora richness, and the Shannon index reflected the diversity of the flora. The *p*-value was obtained from a two-sided test, and *p* < 0.05 indicated statistical significance.

## Results

### Successful establishment of the UR rat model

**Fig. 1:**
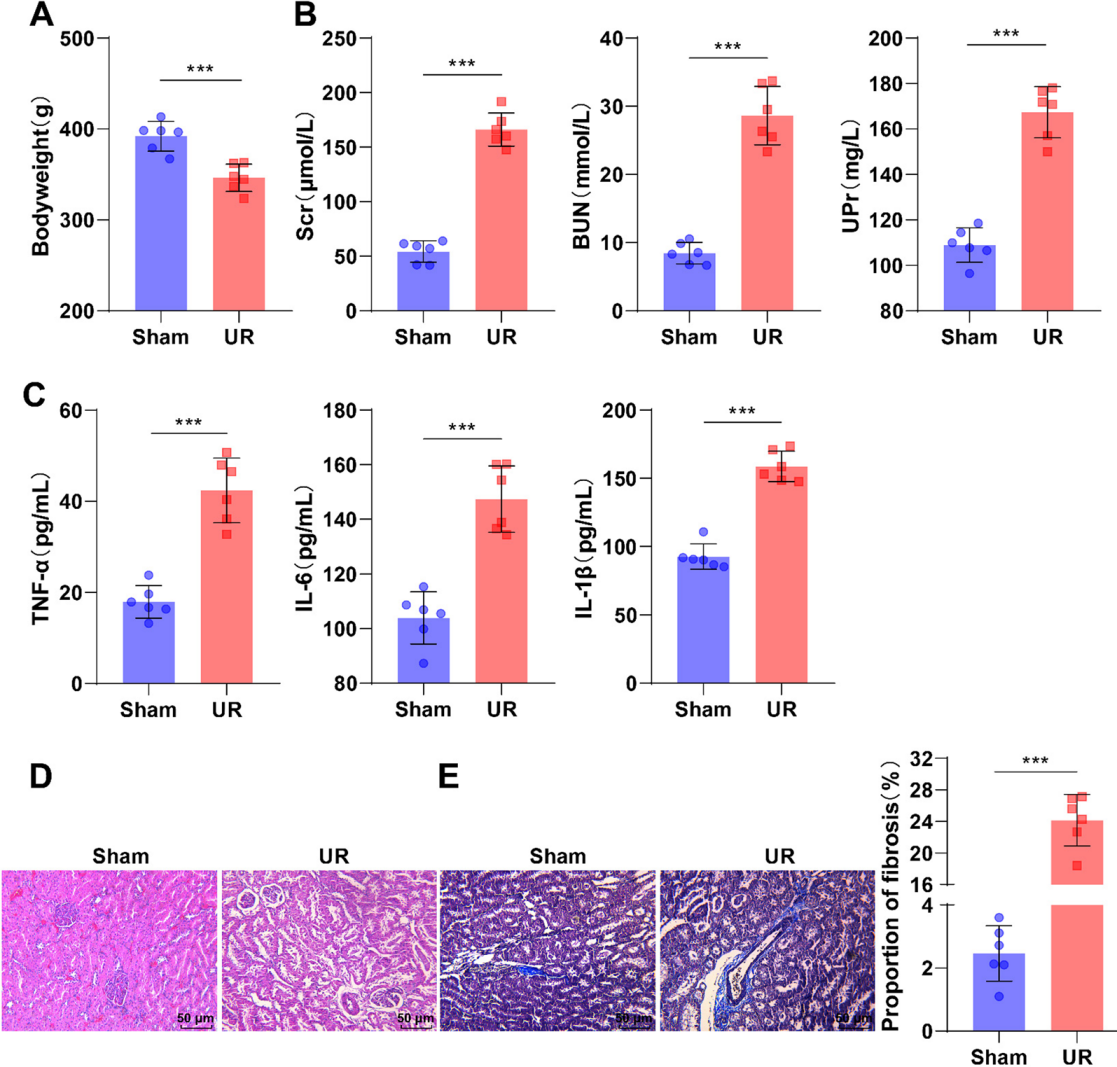
Successful establishment of the UR rat model. **A** Body weight. **B** ELISA detection of Scr, BUN, and UPr levels. **C** ELISA assessment of serum inflammatory factor TNF-α, IL-6, and IL-β levels. **D** H&E staining to observe pathological changes in renal tissues. **E** Masson staining to observe alterations in renal tissue fibrosis. n = 6. Data were represented as mean ± SD and were inspected using an independent sample* t*-test. ***p < 0.001

Referring to the literature (Chen et al. 2019), a UR rat model was established by classical 5/6 nephrectomy. The body weight of rats in the UR group was decreased, the levels of Scr and BUN were raised by two times, and the levels of UPr, TNF-α, IL-6, and IL-1β were also increased prominently, as compared with the Sham group (Fig. 1A–C, all *p* < 0.001). Regarding the results of H&E and Masson staining, in the Sham group, the renal tubular exhibited a normal structure and compact layout, without any expansion of the interstitium and no inflammatory cell infiltration; however, in the UR group, renal tubules were dilated or contracted, along with interstitial swelling, localized infiltration of inflammatory cells (Fig. 1D), collagen deposition, and conspicuously altered fibrosis (Fig. 1E, p < 0.001). The results suggested that the UR rat model was successfully created.

### The richness and diversity of intestinal flora in UR rats were decreased, along with the unbalanced structure of intestinal flora

**Fig. 2:**
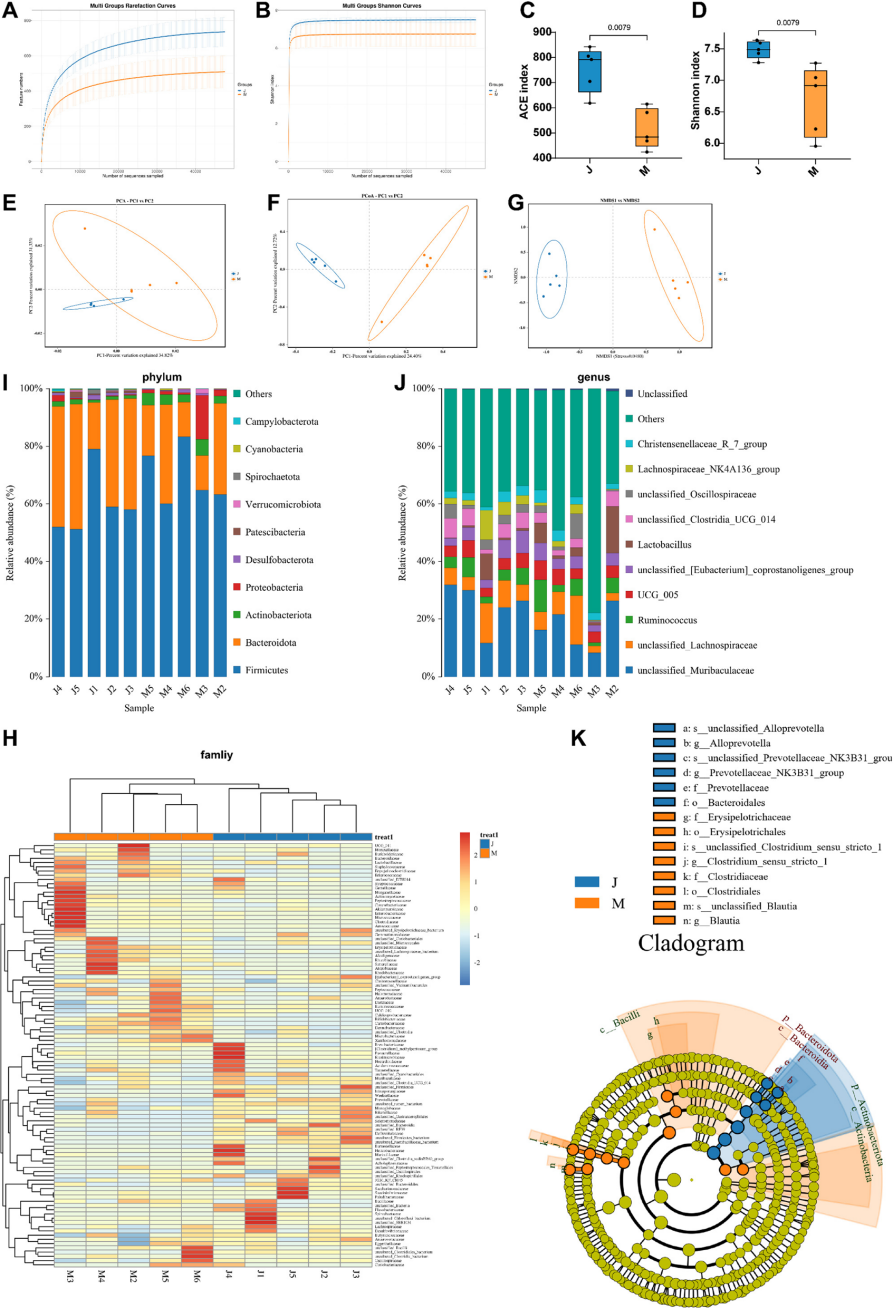
UR rats had reduced intestinal flora richness and diversity, with imbalanced flora structure. **A** Dilution curve; **B** Shannon index curve; **C** ACE index; **D**: Shannon index; **E** PCA analysis; **F** PCoA analysis; **G** Nonmetric multidimensional scaling (NMDS) analysis; **H** Species abundance clustering heatmap (family); **I** Species distribution map (phylum); **J** Species distribution map (genus); **K** LEfSe analysis evolutionary branching diagram. n = 5. Data in panel **C**, **D** were expressed as median (minimum, maximum) and tested using the Mann–Whitney test. “J” denoted the Sham group, and “M” represented the UR group

Firstly, we analyzed the structure of the intestinal flora in rats by 16S rDNA sequencing. The end of the dilution curve and the Shannon index curve gradually flattened, indicating that the sample size was sufficient (Fig. 2A, B). Additionally, the α-diversity analysis presented that the ACE index and Shannon index were lower in the UR group versus the Sham group, suggesting the decline of intestinal flora diversity and richness in UR rats (Fig. 2C, D, all *p* < 0.01). The β-diversity analysis depicted that the composition of rat intestinal flora in the two groups had obvious clustering, and the flora structure was quite different (Fig. 2E–I). Phylum-level analysis revealed that the gut flora was primarily composed of Firmicutes and Bacteroidota, which accounted for the largest proportion. (Fig. 2I). The analysis of genus level unraveled that the dominant bacteria with high relative abundance comprised UCG_005, unclassified_Lachnospiraceae, Ruminococcus, unclassified_[Eubacterium]_coprostanoligenes_group, unclassified_Muribaculaceae, and Lactobacillus (Fig. 2J). The TOP6 bacteria in the relative abundance of the Sham group were unclassified_Alloprevotella, g_Alloprevotella, unclassified_Prevotellaceae_NK3B31_group, Prevotellaceae_NK3B31_group, f_Prevotellaceae, and o_Bacteroidales. The TOP8 bacteria regarding relative abundance in the UR group were f_Erysipelotrichaceae, o_Erysipelotrichales, s_unclassified_Clostridium_sensu_stricto_1, g_Clostridium_sensu_stricto_1, f_Clostridiaceae, o_Clostridiales, s_unclassified_Blautia, and g_Blautia (Fig. 2K). Collectively, the gut microbiota richness and diversity of UR rats exhibited a downward trend, and the flora structure was imbalanced.

### Fecal metabolic spectrum analysis in UR rats

**Fig. 3:**
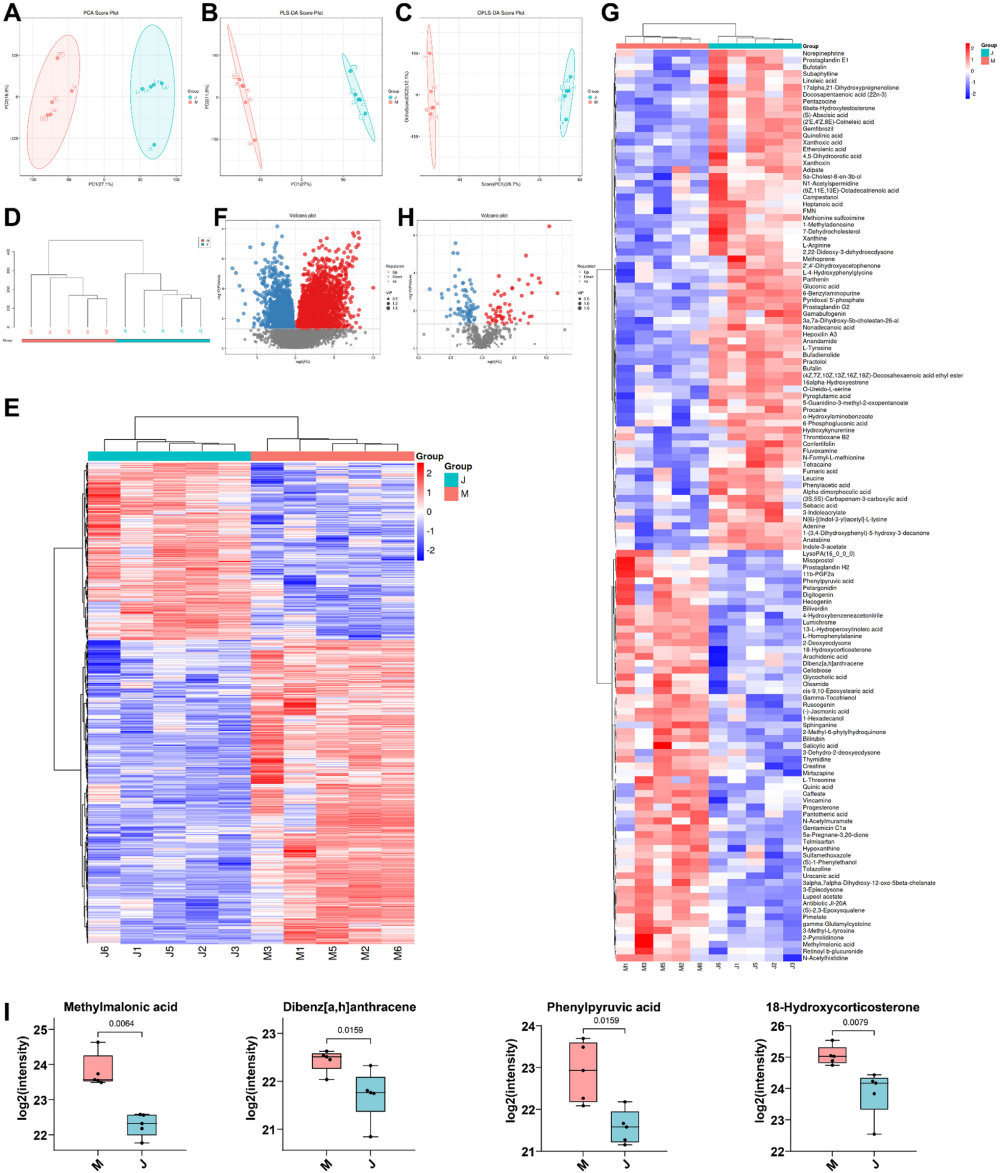
Metabolic spectrum analysis of UR rat feces. **A** PCA analysis; **B** PLS-DA analysis; **C** OPLS-DA analysis; **D** Sample level clustering analysis; **E** Primary differential metabolite clustering heatmap; **F** Volcano plot of primary differential metabolites; **G** Secondary differential metabolite clustering heatmap; **H** Volcano plot of secondary differential metabolites; **I** Analysis of toxin concentration in feces. n = 5. Data in panel **I** were expressed as median (minimum, maximum) and tested using the Mann–Whitney U test. “J” denoted the Sham group, and “M” represented the UR group

An analysis of the fecal metabolic profile of UR rats was conducted using fecal metabolomics. The results of the PCA analysis manifested that there was a notable difference in the metabolome composition between UR rats and Sham rats (Fig. 3A). The results of PLS-DA and OPLS-DA analyses elicited that the samples between the Sham group and the UR group were visibly separated, and the samples within groups were clustered, elucidating that the differences between the groups were greater than that within groups, and the results were reliable (Fig. 3B, C). Afterward, varying metabolites between the two groups were normalized for cluster analysis. A total of 3054 first-order differential metabolites (2220 up-regulated and 834 down-regulated) and 133s-order differential metabolites (60 up-regulated and 73 down-regulated) were screened (Fig. 3D–H). Importantly, the concentrations of MMA (*p* = 0.0064), Dibenz [a, h] anthracene (*p* = 0.0159), Phenylpyruvic acid (*p* = 0.0159), and 18-Hydroxycorticosterone (*p* = 0.0079) in the feces of rats were substantially higher in the UR group than in the Sham group (Fig. 3I). As a result, we chose the most significantly different enterogenous toxin, MMA, as our follow-up study subject.

### Enterogenous toxin MMA facilitated intestinal barrier impairment and increased intestinal permeability in UR rats

**Fig. 4:**
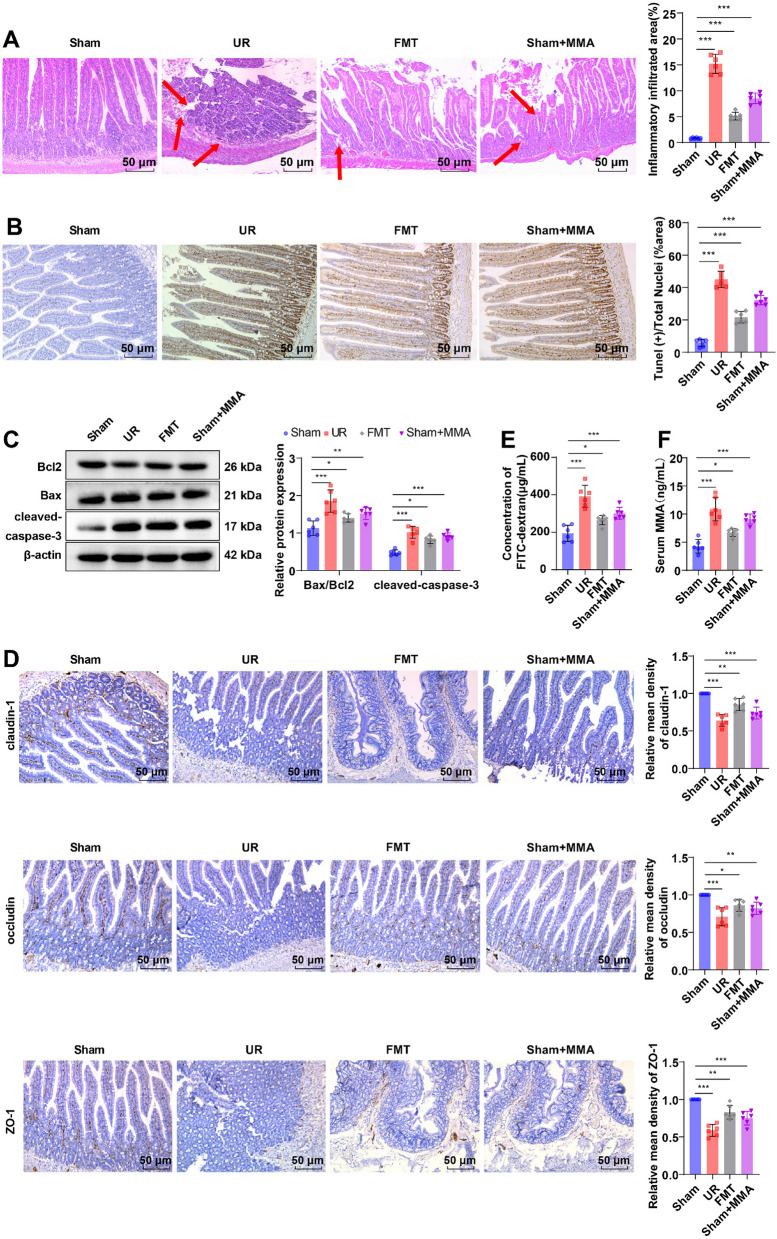
Enterogenous toxin MMA aggravated intestinal barrier impairment and increased intestinal permeability. **A** Observation of colon tissue pathological changes by H&E staining. The red arrows indicated the lesion site; **B** Evaluation of colon tissue cell apoptotic rate with the TUNEL assay kit; **C** Detection of Bax/Bcl2 and cleaved-caspase-3 protein expression levels in the colon by western blot; **D** Assessment of the expression levels of claudin-1, occludin, and ZO-1 in the colon by IHC; **E** Evaluation of intestinal permeability by fluorescence FITC-glucan method; **F** Determination of serum MMA concentration using a reagent kit. n = 6. Data were exhibited as mean ± SD. One-way ANOVA was used for comparisons between groups, and Tukey’s multiple comparison test was used afterward. *p < 0.05, **p < 0.01, ***p < 0.001

To explore whether the changes in intestinal flora and its metabolite MMA affected the occurrence of UR, we transplanted the fecal bacteria of UR rats to Sham rats. Concerning the results of H&E staining, the colon tissue structure of rats in the Sham group was complete, with well-arranged intestinal glands, whereas the colon tissue structure in the UR and FMT groups was destroyed, with a reduced number of intestinal glands and notably augmented area of local inflammatory cell infiltration (Fig. 4A, all *p* < 0.001). In addition, in contrast to the Sham group, the apoptotic rate and the levels of apoptosis-related proteins B-cell lymphoma-2 (Bcl2), Bcl-2-associated X (Bax), and cleaved-caspase-3 in the UR group and the FMT group were markedly up-regulated (Fig. 4B, C, all *p* < 0.05), the expression levels of tight junction proteins claudin-1, occludin, and zonula occludens-1 (ZO-1) were strikingly down-regulated, and the content of FITC-glucan in the blood was distinctly elevated (Fig. 4D, E, all *p* < 0.05). These results demonstrated that some substances in the fecal bacteria of UR rats contributed to intestinal barrier damage and increased intestinal permeability in rats. We selected the MMA with the most significant differences in the fecal metabolome for our study and injected MMA into Sham rats in vitro. The results elicited that serum MMA concentration was markedly higher in the UR, FMT, and Sham + MMA groups than in the Sham group (Fig. 4F, all *p* < 0.01). Furthermore, we analyzed the structure and function of the intestinal barrier in rats. Compared with the Sham group, the Sham + MMA group displayed intestinal barrier damage and increased intestinal permeability (Fig. 4A–E, all *p* < 0.01).

### Enterogenous toxin MMA led to glomerular podocyte loss and lowered GFR, causing calcium-phosphorus metabolic disorder in UR rats

**Fig. 5:**
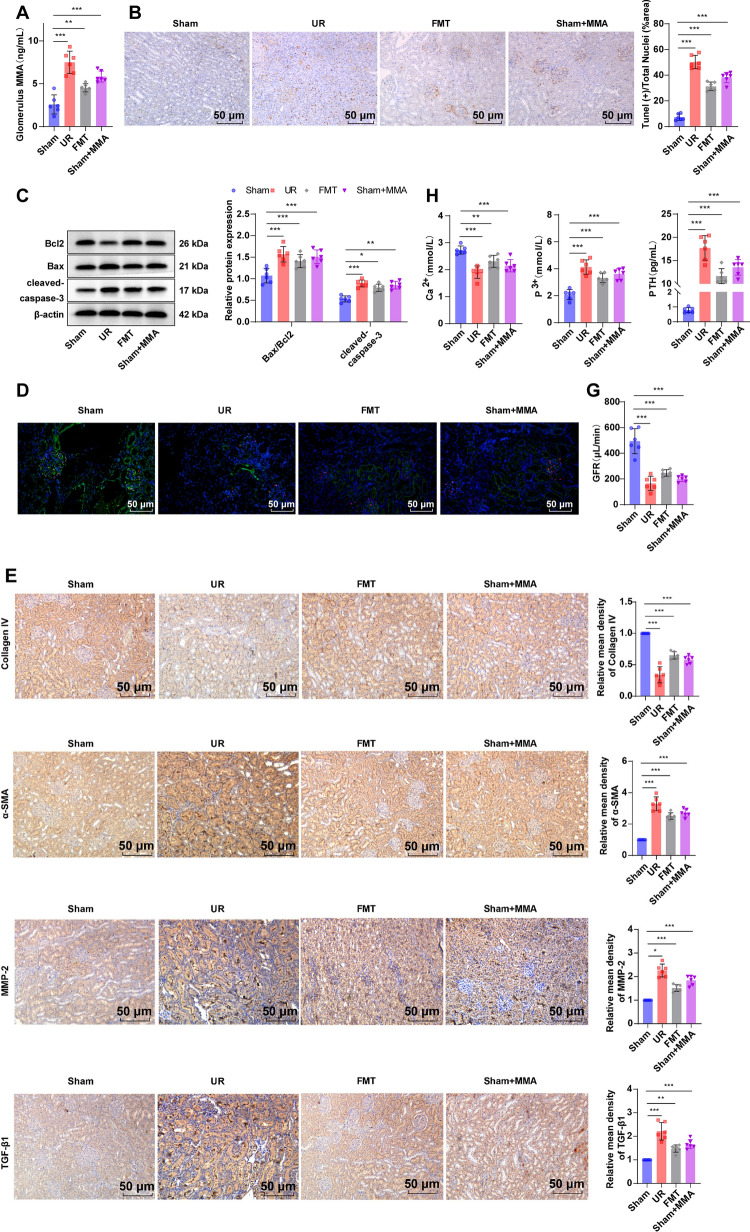

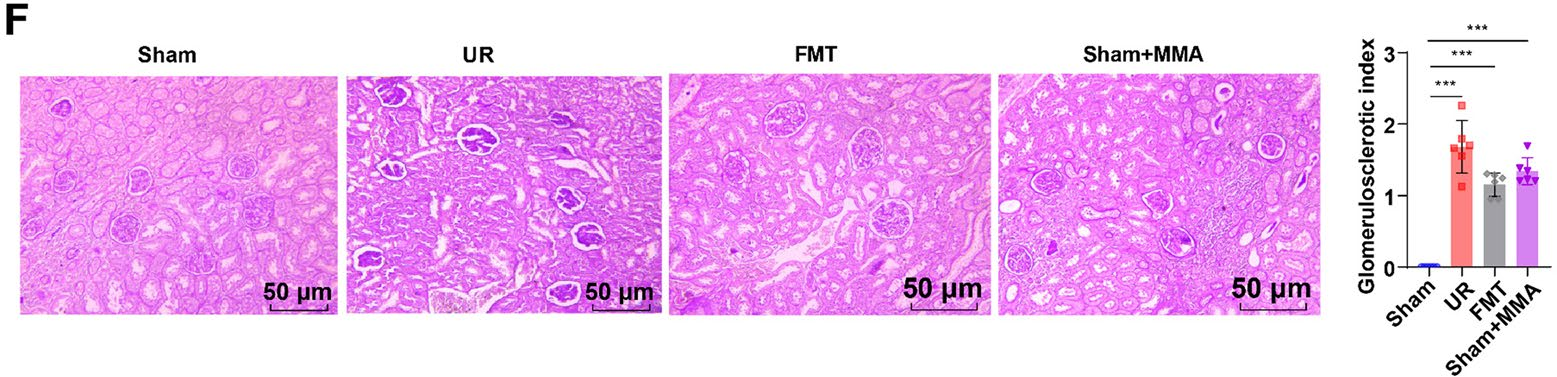
Enterogenous toxin MMA provoked glomerular podocyte loss and diminished GFR, thereby triggering calcium-phosphorus metabolic disorder in UR rats. **A** Detection of MMA concentration in renal tissues using a reagent kit; **B** Examination of renal tissue cell apoptotic rate using TUNEL assay kit; **C** Determination of Bax/Bcl2 and cleaved-caspase-3 protein expression patterns in renal tissues by western blot; **D** Immunofluorescence detection of expression patterns of podocyte-specific markers WT-1 and PODXL; **E** Measurement of Collagen IV, MMP-2, α-SMA and TGF-β1 expression levels in renal tissues by IHC; **F** Calculation of glomerulosclerosis index by PAS staining; **G** Determination of GFR using FITC-inulin clearance; **H** Test of serum Ca^2+^, P^3+^, and PTH levels using a fully automatic biochemical analyzer and an electrochemiluminescence immunoassay analyzer. n = 6. Data were expressed as mean ± SD, Comparisons between multiple groups were analyzed by one-way ANOVA, followed by Tukey’s multiple comparison test. *p < 0.05, **p < 0.01, ***p < 0.001

UR is primarily brought on by podocyte loss (Artelt et al. 2018). Subsequently, we probed whether UR rat feces and enterogenous toxin MMA were absorbed into the blood circulation through the impaired intestinal barrier to further induce glomerular podocyte loss and calcium-phosphorus metabolic disorder. In comparison to the Sham group, the MMA concentration, apoptotic rate, and levels of apoptosis-related proteins Bax/Bcl2 and cleaved-caspase-3 in the UR, FMT, and Sham + MMA groups were considerably up-regulated (Fig. 5A–C, all *p* < 0.05), the expression patterns of WT-1 and podocalyxin (PODXL) (Fig. 5D) and Collagen IV were prominently diminished, and MMP-2, α-smooth muscle actin (α-SMA) and transforming growth factor-β1 (TGF-β1) expression levels were augmented (Fig. 5E, all *p* < 0.01). UR rats in the UR, FMT, and Sham + MMA groups had notably higher glomerulosclerosis index than the Sham group (Fig. 5F, all *p* < 0.01). Furthermore, there were dramatically lessened GFR and serum Ca^2+^, and elevated serum P^3+^ and PTH levels in the UR, FMT, and Sham + MMA groups versus the Sham group (Fig. 5G, H, all *p* < 0.001). Overall, enterogenous toxin MMA stimulated a loss of glomerular podocytes and a decline of GFR, thus causing a calcium-phosphorus metabolic disorder in rats.

### Enterogenous toxin MMA triggered inflammatory responses and boosted apoptosis of rat glomerular podocytes by activating the Wnt/β-catenin pathway

**Fig. 6:**
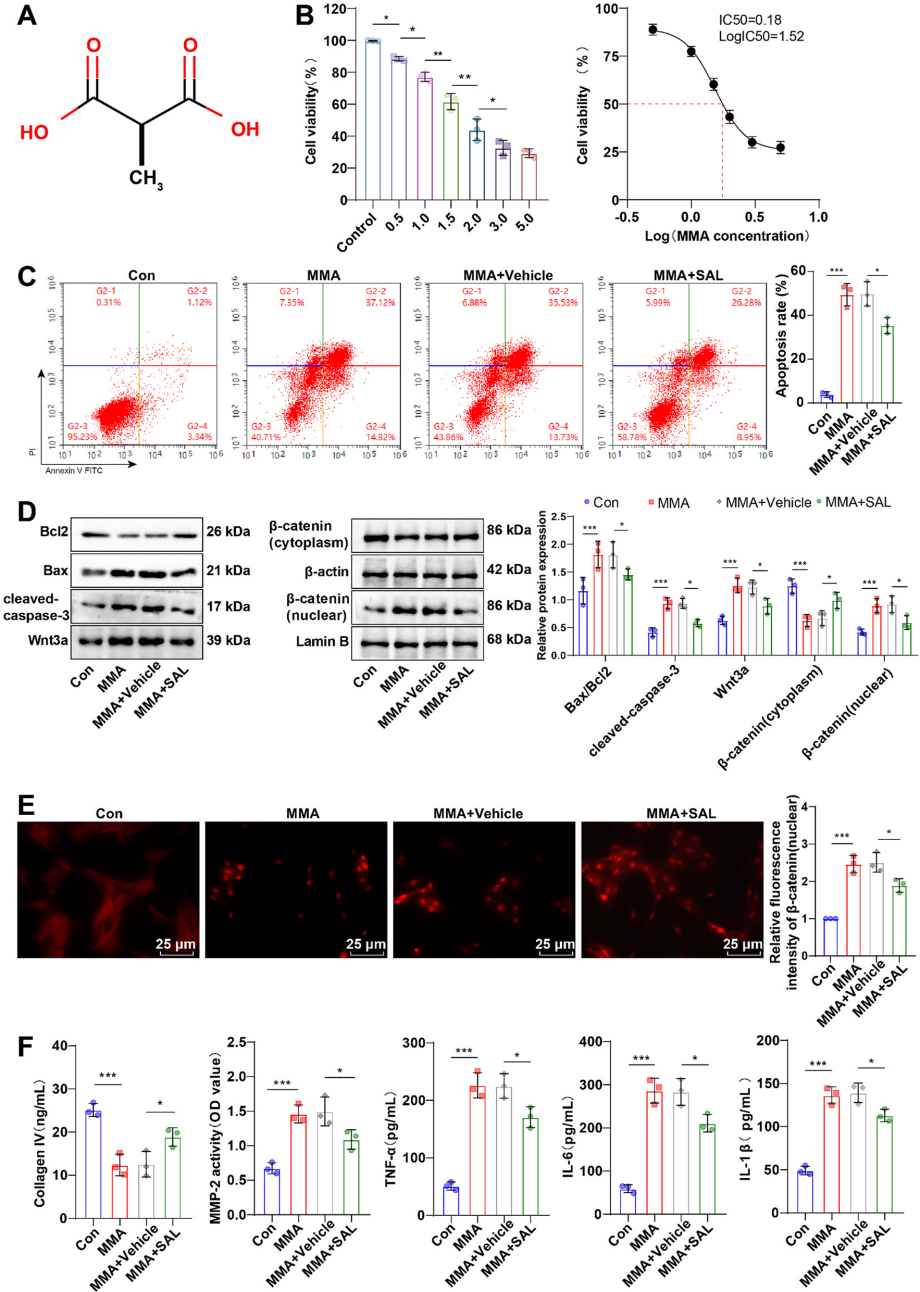
Enterogenous MMA caused inflammatory responses and enhanced apoptosis in rat glomerular podocytes by stimulating the Wnt/β-catenin pathway. **A** MMA chemical structure; **B** MTT assay to assess cell viability and calculate the IC50 value; **C** Cell apoptosis was evaluated by flow cytometry. **D** Western blot to determine the protein expression levels of Bax/Bcl2, cleaved-caspase-3, Wnt3a, β-catenin (nuclear) and β-catenin (cytoplasmic); **E** Immunofluorescence to measure the nuclear translocation level of β-catenin; **F** The expression levels of Collagen IV, MMP-2, TNF-α, IL-6 and IL-1β were examined using kits. The cell experiment was repeated three times independently. Data were denoted as mean ± SD. One-way ANOVA was implemented for comparisons among groups, and Tukey’s multiple comparisons test was used afterward. *p < 0.05, **p < 0.01, ***p < 0.001

To investigate whether MMA stimulated inflammatory responses and enhanced rat glomerular podocyte apoptosis by modulating the Wnt/β-catenin pathway, we cultured rat glomerular podocytes in vitro and treated cells with different doses of MMA (0.5, 1.0, 1.5, 2.0, 3.0, and 5.0 mmol/L) for 24 h. The chemical structure of MMA is illustrated in Fig. 6A. Based on the MTT assay, the cell viability declined dose-dependently in response to treatment with medium-to-low dosages of MMA (0.5–3.0 mmol/L) (Fig. 6B, all *p* < 0.05), while there was no apparent difference in cell viability between cells treated with high-dose MMA (5.0 mmol/L) and cells treated with 3.0 mmol/L MMA (Fig. 6B, *p* > 0.05). The IC50 value of MMA-treated rat glomerular podocytes was calculated to be 1.52 mmol/L using the GraphPad Prism 9.5.0 software (Fig. 6B). Therefore, we used 1.5 mmol/L MMA to treat cells in subsequent experiments. The apoptotic rate and levels of Bax/Bcl2 and cleaved-caspase-3 in the MMA group were appreciably higher than those in the Control group (Fig. 6C, D, all *p* < 0.001). Besides, in comparison with the Control group, Collagen IV levels in the MMA group dropped evidently, and TNF-α, MMP-2, IL-6, and IL-1β levels conspicuously rose (Fig. 6F, all *p* < 0.001). Beyond that, the Wnt3a/β-catenin (nuclear) protein expression and β-catenin nuclear translocation level in the MMA group was remarkedly up-regulated, and the β-catenin (cytoplasmic) protein was notably down-regulated (Fig. 6D, E, all *p* < 0.001). Following this, we added 10 μM SAL (the Wnt/β-catenin pathway inhibitor) to MMA-treated cells and found no evident difference in any of the indexes between the MMA group and the MMA + Vehicle group (Fig. 6C–F, all *p* > 0.05). Notwithstanding, compared with the MMA + Vehicle group, the MMA + SAL group exhibited prominently up-regulated β-catenin (cytoplasmic) protein and Collagen IV levels, together with noticeably down-regulated levels of β-catenin nuclear translocation, cleaved-caspase-3 and Bax/Bcl2, MMP-2, TNF-α, IL-6 and IL-1β, Wnt3a/β-catenin (nuclear) protein expression, and cell apoptotic rate (Fig. 6C–F, all *p* < 0.05). In short, these findings suggested that enterogenous toxin MMA activated the Wnt/β-catenin pathway, provoked inflammatory responses, and expedited apoptosis of rat glomerular podocytes.

### Blocking the Wnt/β-catenin pathway partially reversed the inducing effect of enterogenous toxin MMA on the calcium-phosphorus metabolic disorder in UR rats

**Fig. 7:**
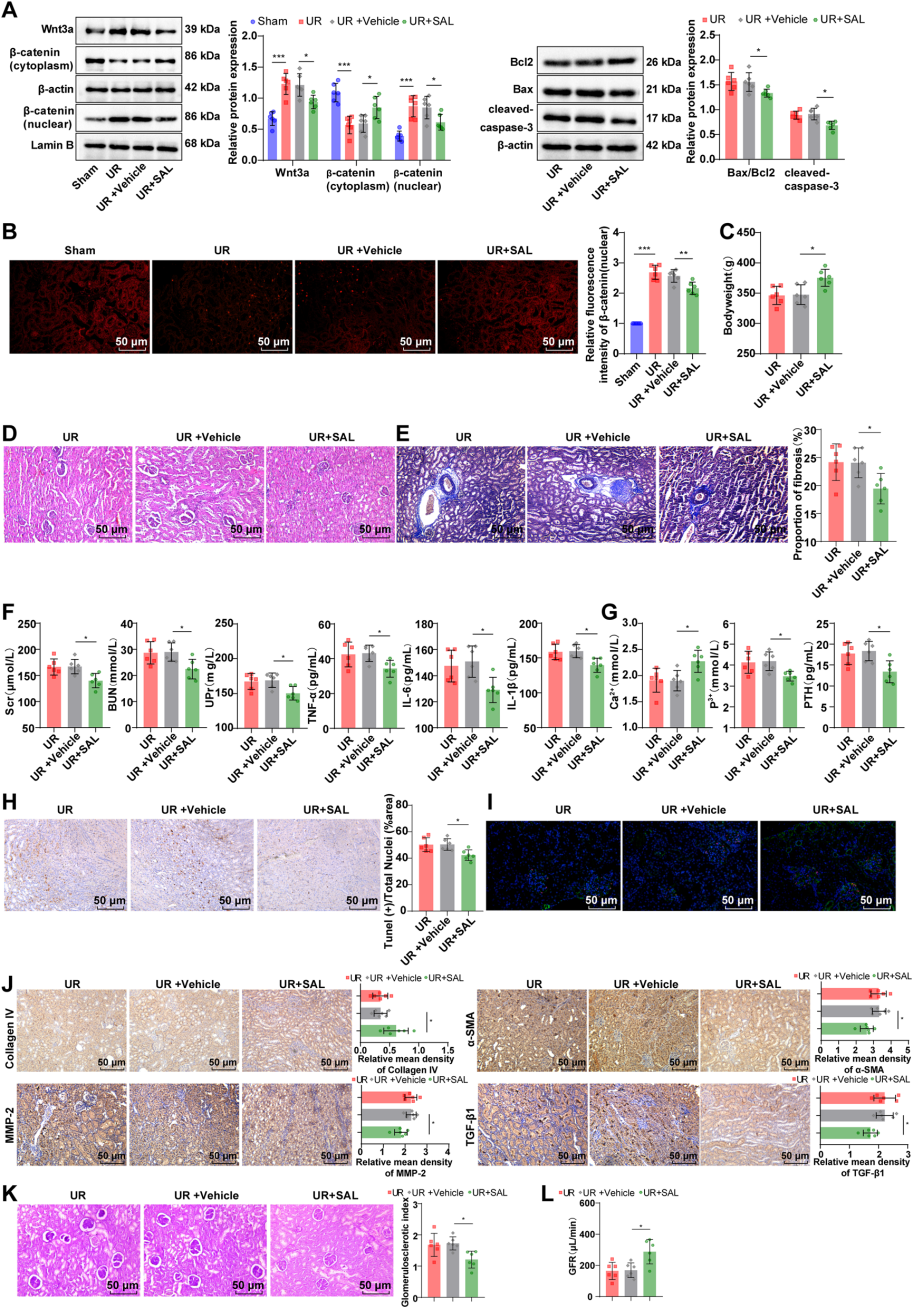
Impeding the Wnt/β-catenin pathway partially nullified the inducing effect of enterogenous toxin MMA on the calcium-phosphorus metabolic disorder in UR rats. **A** Western blot to determine Wnt3a, β-catenin (nucleus), β-catenin (cytoplasm), Bax/Bcl2, and cleaved-caspase-3 protein expression levels; **B** Immunofluorescence to measure β-catenin nuclear translocation level; **C** Body weight; **D** H&E staining to observe pathological alterations in renal tissues; **E** Masson staining to observe changes in renal tissue fibrosis; **F** Determination of Scr, BUN, UPr, serum TNF-α, IL-6 and IL-β levels by ELISA; **G** The serum Ca^2+^ and P^3+^ levels were assessed using a fully automated biochemical analyzer, and the serum PTH level was determined using an electrochemiluminescence immunoassay analyzer; **H** Evaluation of renal tissue cell apoptotic rate using TUNEL assay kit; **I** Examination of WT-1 and PODXL expression levels by immunofluorescence; **J** IHC to measure Collagen IV, MMP-2 α-SMA and TGF-β1 expression patterns in renal tissues; **K** Calculation of glomerulosclerosis indexes by PAS staining; **L** Determination of GFR by FITC-inulin clearance. n = 6. Data were presented as mean ± SD. One-way ANOVA was performed for comparisons among groups, followed by Tukey’s multiple comparison test. *p < 0.05, ***p < 0.001

To validate the inductive effect of the Wnt/β-catenin pathway on MMA-mediated calcium-phosphorus metabolic disorder in UR rats, we intraperitoneally injected 8 mg/kg SAL into 5/6 nephrectomy-operated rats. The UR group displayed noticeably raised Wnt3a, β-catenin (nuclear) protein, and β-catenin nuclear translocation, as well as diminished β-catenin (cytoplasmic) protein relative to the Sham group (Fig. 7A, B, all *p* < 0.001); yet, the levels of β-catenin (nuclear) protein, Wnt3a, and β-catenin nuclear translocation were down-regulated, and β-catenin (cytoplasmic) protein was up-regulated in the UR + SAL group versus the UR + Vehicle group (Fig. 7A, B, all *p* < 0.05). There was no significant distinction in each index between the UR group and the UR + Vehicle group (Fig. 7A–L, all *p* > 0.05). Additionally, in contrast to the UR + Vehicle group, rats in the UR + SAL group had raised body weight, serum Ca^2+^ level and GFR, ameliorative renal tubular acidosis, and reduced local inflammatory cell infiltration, collagen deposition, interstitial fibrosis degree, podocyte loss, glomerulosclerosis index, and renal tissue cell apoptotic rate, and diminished levels of cleaved-caspase-3, Bax/Bcl2,, Scr, BUN, UPr, serum TNF-α, IL-6, IL-β, P^3+^ and PTH (Fig. 7A–L, all *p* < 0.05). As a consequence, hindering the Wnt/β-catenin pathway partly averted the inductive effect of enterogenous toxin MMA on the calcium-phosphorus metabolic disorder in UR rats.

## Discussion

CKD is marked by a decrease in the pace at which the kidneys filter blood, an increment in the amount of albumin discharged in urine, and a buildup of various metabolic waste products in the body that are typically eliminated by the kidneys (Jha et al. 2013). The metabolites are referred to as uremic toxins or uremic retention solutes when they accumulate to hazardous levels in the body as a result of UR (Vanholder et al. 2003). Intriguingly, the elevated MMA level is a potential marker of aberrant gut flora, and MMA concentration is linked to renal impairment and endogenous generation of propionic acid (Riphagen et al. 2020). Here, we developed a UR rat model to explore the effect of MMA on calcium-phosphorus metabolic disorder. This present study revealed that the enterogenous toxin MMA exacerbated calcium-phosphorus metabolic disorder in UR rats by activating the Wnt/β-catenin pathway.

There is evidence suggesting that the gut microbiota has a role in the creation of uremic toxins, which worsens the health of CKD patients (Popkov et al. 2022). The intestinal barrier will endure histological changes in response to the stimulation of diseases and toxins (Magnusson et al. 1991). Excessive apoptosis of intestinal epithelial cells can result in harm to the intestinal mucosal barrier and an increase in intestinal permeability (Goncalves et al. 2006; Almeida Duarte et al. 2004). Toxins generated and stored in the intestinal cavity are absorbed into the bloodstream via the intestinal wall, prompting the body to generate inflammatory agents and leading to microinflammation (Raj et al. 2009; McIntyre et al. 2011; Feroze et al. 2012). In the current study, after the successful establishment of the UR rat model, we analyzed the intestinal flora structure and observed suppressed richness and diversity as well as unbalanced structure in UR rats. Consistently, multiple studies have shown that patients with CKD experience notable alterations in their gut microbiota, bringing about a decrease in richness, α-diversity, and β-diversity (Plata et al. 2019; Meijers et al. 2019; Mahmoodpoor et al. 2017). Also, Jialin Hu et al. demonstrated that an imbalanced gut mycobiome may appear in CKD patients, which was also associated with immune system dysfunction (Hu et al. 2022). In a meta-analysis, Stanford et al. described the great dissimilarity between CKD patients and healthy controls in about twenty microbial taxa (Stanford et al. 2020). In addition, we used fecal metabolomics to normalize different metabolites for cluster analysis and found that the toxin MMA concentration in UR rat feces differed significantly from the controls. Therefore, we selected the enterogenous toxin MMA to explore its participation in the intestinal barrier of UR rats.

There is a hypothesis suggesting that an increased level of MMA can potentially harm the kidney (Franques et al. 2017). Moreover, the impairment of the intestinal barrier in animals with CKD is caused by the degradation of claudin-1, occludin, and ZO-1 components of the tight junctions in the epithelial cells (Vaziri et al. 2012a). The UR-specific tight junction permeability barrier is compromised, mostly due to the decreased expression of claudin-1, occludin, and ZO-1 (Vaziri et al. 2012b). Correspondingly, in our study, UR rats exhibited damaged colon tissue structure, local inflammatory cell infiltration, augmented MMA concentration diminished intestinal gland numbers, and repressed levels of ZO-1, claudin-1, and occludin. As reported, elevated levels of MMA may occur unexpectedly in elderly patients, those with renal failure or volume contraction, and those with an overgrowth of intestinal flora (Vogiatzoglou et al. 2009; Tun et al. 2017). Reports regarding serum MMA level and intestinal barrier injury in UR rats were limited, and our study for the first time showed that enterogenous toxin MMA prompted disruption of the intestinal barrier and increased intestinal permeability in rats.

Wiggins put forward the notion that the reduction of podocytes, along with the enlargement of dysfunctional podocytes, had a role in the development of glomerulosclerosis and ongoing proteinuria, ultimately leading to the progression to ESRD (Wiggins 2007). Podocytes adhere to the outer side of the glomerular basement membrane, which, together with the glomerular basement membrane and glomerular basement membrane, constitute the glomerular hemofiltration barrier. Previous studies have indicated that podocyte depletion is strongly associated with glomerulosclerosis and serves as the primary factor contributing to urinary retention (Artelt et al. 2018; Sato et al. 2009). Podocytes participate in the formation of the glomerular filtration barrier (Diez-Sampedro et al. 2011), with the membrane protein podocalyxin playing a crucial role in preserving the integrity of the glomerular filtration function (Vitureira et al. 2010). Podocytes are known to secrete type IV collagen and laminin, which are the primary constituents of the glomerular basement membrane. Additionally, podocytes secrete MMPs, which play a crucial role in maintaining the glomerular basement membrane's metabolic homeostasis by aiding in the degradation of the membrane in response to stimulation of renal fibrosis (Yao et al. 2015). Furthermore, renal fibrosis results in a progressive decline of renal function, ultimately entering the UR stage (Klahr and Morrissey 2002), which is marked by a notable rise in the expression levels of fibroblast markers α-SMA and TGF-β1 (Li et al. 2019), along with the heightened secretion of pro-inflammatory cytokines TNF-α, IL-6, and IL-1β.

Besides, the presence of damaged podocytes in urine sediment has been linked to several urinary biomarkers, including PODXL, nephrin, podocin, and WT-1 (Sekulic and Pichler 2013). PODXL-deficient mice have defective growth of podocyte foot processes, resulting in anuria and renal failure (Barua et al. 2014). Moreover, decreased expression of WT1 can result in lower levels of nephrin and PODXL, which are linked to kidney diseases like glomerulosclerosis (Seo et al. 2015; Mishra et al. 2016; Dong et al. 2015). Also, the rising level of PTH has a connection to the development of cardiac fibrosis and renal impairment in cases of UR (Sobrevia et al. 2011). Notably, disruptions in calcium-phosphorus metabolism arise when there are deviations from the normal amounts of calcium and phosphorus in the body, which can be caused by alterations in calcium, phosphate, and PTH (Padrini 1987). The above data support our findings that UR rats that MMA stimulated the depletion of glomerular podocytes and declined GFR, hence evoking disturbances in calcium and phosphorus metabolism in UR rats. Another interesting finding was that the induction of MMA on calcium-phosphorus metabolism disturbances in UR rats could be partly counteracted by impeding Wnt/β-catenin.

It is interesting to note that enterogenous toxin MMA can stimulate the Wnt/β-catenin pathway (Hu et al. 2023) while blockage of the Wnt/β-catenin signaling safeguards renal function in rats subjected to 5/6 nephrectomy (Mo et al. 2015). Numerous studies confirmed that the Wnt/β-catenin pathway is implicated in renal cell injury, encompassing harm to podocytes, tubular cells, and mesangial cells (Zhou et al. 2012; Senouthai et al. 2019). We found that MMA-treated cells had higher apoptotic rates, and levels of apoptosis-related protein, inflammatory cytokines, Wnt3a, β-Catenin (nuclear) protein expression, and β-Catenin nuclear translocation, as well as lower β-catenin (cytoplasmic) protein, while these alterations could be reversed by the Wnt/β-Catenin pathway inhibitor. Consistently, the activation of the Wnt/β-catenin pathway in DN is bound up with mesangial cell apoptosis and epithelial-mesenchymal transition (Xiao et al. 2013). In contrast, a similar study reported that the inhibition of the Wnt pathway by DKK-1 prompted an improvement in the apoptosis of podocytes (Wang et al. 2021). Moreover, transverse aortic constriction-induced heart injury facilitates systemic inflammation, as evidenced by elevated TNF-α and IL-1β by activating Wnt/β-catenin (Zhao et al. 2019). Consequently, these findings altogether illustrated the promotional effect of regulating Wnt/β-catenin in cell apoptosis and inflammatory reactions in MMA-treated rat glomerular podocytes.

Nevertheless, some limitations of the present study still need to be addressed. First, we did not perform fecal microbiome analysis after the FMT experiment. Second, we did not analyze the correlation between changes in intestinal flora and changes in the MMA level. Third, only male rats, but not female rats, were used in our study, and rats of different sexes might affect calcium and phosphorus metabolism (Matsuzaki et al. 2002). Finally, we only preliminarily explored the mechanism of enterogenous toxin MMA aggravating calcium-phosphorus metabolism disorder in UR rats by regulating the Wnt/β-catenin pathway. In the future, We will perform further fecal microbiome analyses and explore the specific strains that influence changes in the MMA level, and will also add female rats to the experiment to rule out the influence of the sex factor on metabolism. We will further perform RNA sequencing KEGG pathway enrichment analysis on glomerular podocytes treated with MMA to elucidate the mechanism by which MMA exacerbates calcium-phosphorus metabolic abnormalities in UR rats. Additionally, on the basis of our research, we will explore new strategies to modulate the homeostasis of intestinal flora in the treatment of CKD, such as FMT in UR rats using beneficial bacteria against the enterogenous toxin MMA, and re-use 16S rDNA sequencing and metabolomics to analyze the intestinal flora structure and fecal metabolic spectrum of rats.

## Conclusions

This study innovatively elucidated that enterogenous toxin MMA promoted intestinal barrier impairment in UR rats, enhanced intestinal permeability, and activated Wnt/β-catenin to induce glomerular podocyte loss and reduce GFR, and then aggravated calcium and phosphorus metabolic disorder, which offered a theoretical foundation for understanding the development of UR and presented novel insights for UR treatment. In the past few decades, intestinal flora has become a key factor in the study of kidney disease. Alterations in the composition of gut microbiota can lead to alterations in the metabolites generated by intestinal bacteria, and imbalanced uremic toxins are the primary contributors to the decline in kidney function (Xie et al. 2022; Caggiano et al. 2023). Our study revealed that fecal transplantation in UR rats aroused elevated serum and renal tissue MMA concentration in Sham rats, whereas MMA further led to renal injury. Controlling intestinal homeostasis is a novel approach and technique for treating CKD, and researchers have extensively investigated the potential of modifying intestinal flora or its metabolites in the management of CKD (Hobby et al. 2019). Our findings may provide new ideas for the treatment of CKD.

## Supplementary Information

Supplementary Material 1: Fig. S1 Experimental flowchart.

Supplementary Material 2.

## Data Availability

All data included in this study are available upon request by contact with the corresponding author.

## Abbreviations

MMA: Methylmalonic acid
UR: Uremia
ESRD: End-stage renal disease
CKD: Chronic kidney disease
H&E: Hematoxylin and eosin staining
PAS: Periodic acid-Schiff staining
ELSA: Enzyme-linked immunosorbent assay
Scr: Serum creatinine
UPr: Urinary protein
TNF-α: Tumor necrosis factor-α
IL-6: Interleukin-6
PTH: Hormone
GFR: Glomerular filtration rate
ZO-1: Zonula occludens-1
MMP-2: Matrix metalloproteinase-2
α-SMA: α-Smooth muscle actin
TGF-β1: Transforming growth factor-β1
WT-1: Wilms tumor 1
PODXL: Podocalyxin
Bcl2: B-cell lymphoma-2
Bax: Bcl-2-associated X
MTT: 4,5-Dimethyl-2-thiazolyl-2,5-diphenyl-2-*H*-tetrazolium bromide
FITC-dextran: Fluorescein isothiocyanate-dextran 4 kDa
SAL: Salinomycin
ASV: Amplicon sequence variant
PCA: Principal component analysis
PLS-DA: Partial least squares discriminant analysis
VIP: Variable projection importance
TUNEL: Terminal deoxynocleotidyl Transfer Mediated Nick End Labeling
OD: Optical density
DN: Diabetic nephropathy
FMT: Fecal microbiota transplantation
FACS: Fluorescence-activated cell sorting
