# Supplementary figures and images for: The mechanism of enterogenous toxin methylmalonic acid aggravating calcium-phosphorus metabolic disorder in uremic rats by regulating the Wnt/β-catenin pathway
Source: Mol Med. 2025 Jan 22;31:19. doi: 10.1186/s10020-025-01067-y (PMC11756144; doi:10.1186/s10020-025-01067-y)

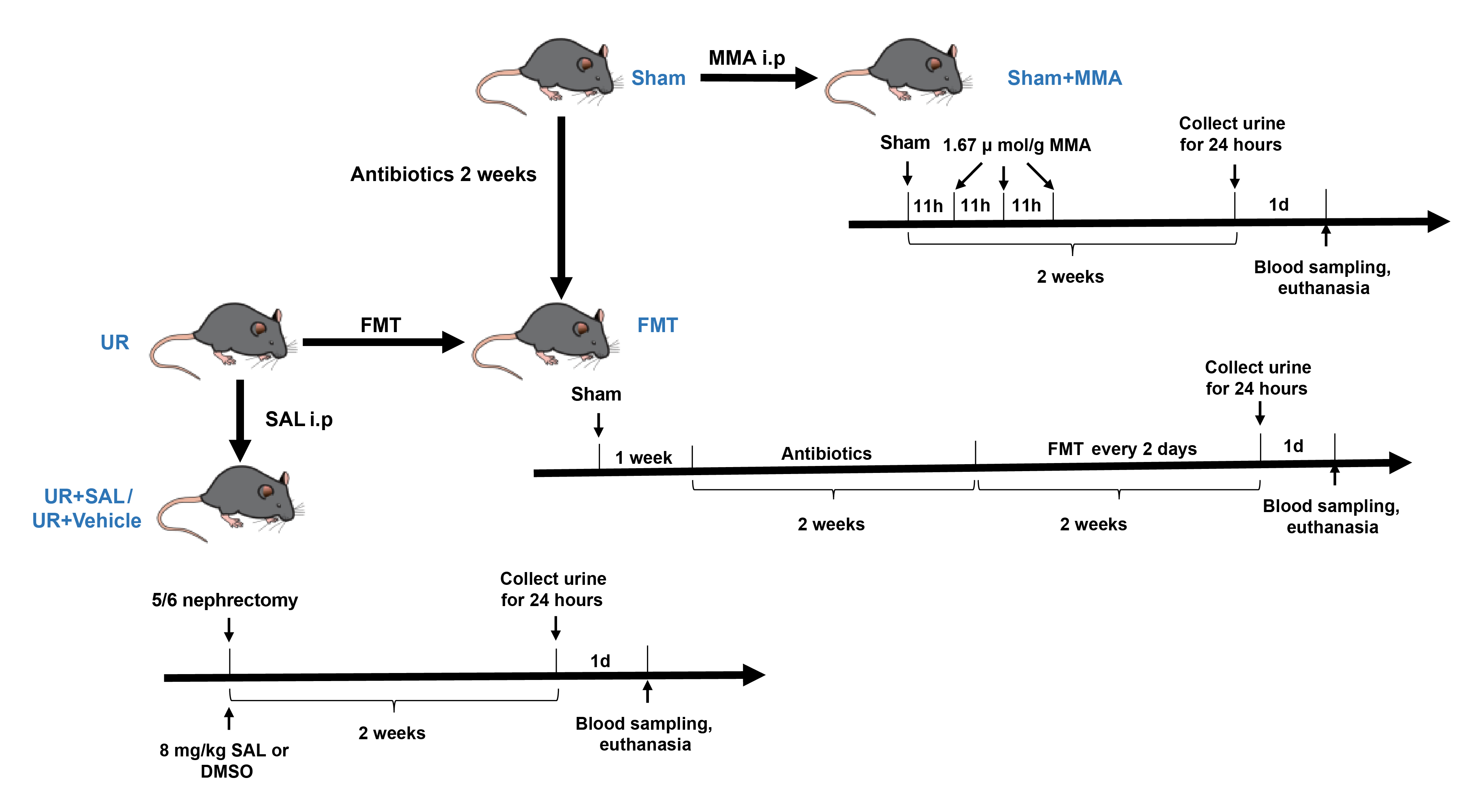

Supplement: Supplementary file 1 — Supplementary Material 1: Fig. S1 Experimental flowchart. [file 10020_2025_1067_MOESM1_ESM.tiff]
