# Supplementary material for: The mechanism of enterogenous toxin methylmalonic acid aggravating calcium-phosphorus metabolic disorder in uremic rats by regulating the Wnt/β-catenin pathway
Source: Mol Med. 2025 Jan 22;31:19. doi: 10.1186/s10020-025-01067-y (PMC11756144; doi:10.1186/s10020-025-01067-y)
Supplement: Supplementary file 2 — Supplementary Material 2. [file 10020_2025_1067_MOESM2_ESM.docx]

**Supplementary Table 1** Antibody related information

| Application | Primary antibodies | Cat No. | Dilution | Company |
| --- | --- | --- | --- | --- |
| Immunohistochemistry | claudin-1 | ab307692 | 1:100 | Abcam |
|  | occludin | ab216327 | 1:200 | Abcam |
|  | ZO-1 | ab221547 | 1:500 | Abcam |
|  | Collagen IV | ab6586 | 1:100 | Abcam |
|  | MMP-2 | ab86607 | 1:100 | Abcam |
|  | α-SMA | ab124964 | 1:1000 | Abcam |
|  | TGF-β1 | ab215715 | 1:500 | Abcam |
| Immunofluorescence | Wilms tumor 1 (WT-1) | GTX131203 | 1:100 | GeneTex |
|  | Podocalyxin (PODXL) | GTX34977 | 1:100 | GeneTex |
|  | β-catenin | ab32572 | 1:250 | Abcam |
| Western blot | cleaved-caspase-3 | GTX86952 | 1:1000 | GeneTex |
|  | Bax | ab32503 | 1:1000 | Abcam |
|  | Bcl2 | ab194583 | 1:1000 | Abcam |
|  | Wnt3a | ab219412 | 1:1000 | Abcam |
|  | β-catenin | ab32572 | 1:5000 | Abcam |
|  | β-actin | ab8227 | 1:1000 | Abcam |
|  | Lamin B | ab16048 | 1:1000 | Abcam |
| Application | Secondary antibodies | Cat No. | Dilution | Company |
| Immunohistochemistry/Western blot | Goat Anti-Rabbit IgG H&L (HRP) | ab6721 | 1:1000 | Abcam |
| Immunofluorescence | Goat Anti-Rabbit IgG (DyLight594) | GTX213110-05 | 1:100 | GeneTex |
|  | Goat Anti-Mouse IgG (DyLight488) | GTX213111-04 | 1:100 | GeneTex |
